# Circular Sponge against miR-21 Enhances the Antitumor Activity of Doxorubicin against Breast Cancer Cells

**DOI:** 10.3390/ijms232314803

**Published:** 2022-11-26

**Authors:** Ana R. Rama, Patricia Lara, Cristina Mesas, Francisco Quiñonero, Celia Vélez, Consolación Melguizo, Jose Prados

**Affiliations:** 1 Department of Health Sciences, University of Jaén, 23071 Jaén, Spain; 2 Institute of Biopathology and Regenerative Medicine (IBIMER), Center of Biomedical Research (CIBM), University of Granada, 18100 Granada, Spain; 3 Institute of Biosanitary Research from Granada (ibs. GRANADA), 18014 Granada, Spain; 4 Department of Anatomy and Embryology, Faculty of Medicine, University of Granada, 18016 Granada, Spain

**Keywords:** miRNA, circular sponges, miR-21, doxorubicin, breast cancer

## Abstract

Breast cancer is the most common type of cancer in women, with chemotherapy being the main strategy. However, its effectiveness is reduced by drug resistance mechanisms. miR-21 is upregulated in breast cancer that has been linked to drug resistance and carcinogenic processes. Our aim was to capture miR-21 with a circular sponge (Circ-21) and thus inhibit the carcinogenic processes and drug resistance mechanisms in which it participates. Proliferation, migration, colony formation, cell cycle, and poly [ADP-ribose] polymerase 1 (PARP-1) and vascular endothelial growth factor (VEGF) detection assays were performed with MCF7 breast cancer cells and MCF10A non-tumor cells. In addition, doxorubicin resistance tests and detection of drug resistance gene expression were performed in MCF7 cells. Reduction in proliferation, as well as migration and colony formation, increased PARP-1 expression, inhibition of VEGF expression and cell cycle arrest in G2/M phase were displayed in the Circ-21 MCF7, which were not observed in the MCF10A cells. Furthermore, in the MCF7 cells, the Circ-21 enhanced the antitumor activity of doxorubicin and decreased the expression of resistance genes: *ABCA1, ABCC4*, and *ABCC5*. Based on these results, the use of Circ-21 can be considered a first step for the establishment of an effective gene therapy in the treatment of breast cancer.

## 1. Introduction

Breast cancer is the most common cancer in women, as well as being the leading cause of cancer death among women worldwide [1]. Chemotherapy is used as the main strategy for its treatment, with doxorubicin (DOXO) being one of the main chemotherapeutic agents used [2]. However, DOXO treatment can affect non-cancerous tissues and cause damage to healthy organs such as the heart, liver, or kidneys [3]. 

Another obstacle in the treatment of breast cancer is resistance to chemotherapy [2,4], which can be caused by several mechanisms, the most relevant being the increase in drug efflux mediated by transporters of the ATP-binding cassette (ABC) [5,6]. These are intra- and extracellular proteins that cross the membrane transporting a wide variety of substrates. The human *ABC* gene family comprises 48 functional transporters distributed in seven subfamilies. In recent years, multiple genetic variations have been found in the *ABC* genes, leading to these differences in breast cancer treatment response and toxicity. [7].

The *ABCA1* gene encodes a transmembrane protein expressed in multiple tissues with different functions. However, its most studied function is the release of intracellular cholesterol and phospholipids through the plasma membrane to combine with apolipoproteins (mainly apolipoprotein AI (ApoA-I)) [8]. The high expression of *ABCA1* is a marker for high-grade triple-negative breast cancer and is associated with an increased risk of tumor recurrence [9]. 

The *ABCC4* gene encodes multidrug resistance protein 4 (MRP4). It is overexpressed in tumor tissues and is capable of effluxing several different chemotherapeutic drugs out of cancer cells [5,10]. *ABCC4* expression is similarly upregulated in chemotherapy-treated breast tumors compared to noncancerous tissue [11]. Additionally, MRP4 may play a role in cancer development and progression due to its ability to transport numerous physiological substrates and its roles in inflammation and metabolism [10,11]. 

On the other hand, the *ABCC5* gene encodes the multi-drug resistance protein 5 protein (MRP5). It contributes to drug resistance and it is involved in the release of various types of anticancer drugs from the cell, such as 6-mercaptopurine (6-MP), 6-thioguanine (6-TG), 5-fluorouracil (5-FU) and their metabolites [12,13]. Moreover, *ABCC5* overexpression has been related with breast cancer and patients with worse prognosis [14,15]. 

miRNA-21 (miR-21) is related to drug resistance and to carcinogenic processes such as processes of cell proliferation, migration and apoptosis [16]. miR-21 is located on chromosome 17 (17q.23.1) in intron 11 of the TMEM 49 gene (transmembrane protein 49), precursor of vacuole membrane protein 1 (VMP1) [17,18]. Nevertheless, miR-21 is upregulated in various types of cancer such as lung, ovary, breast, stomach, prostate, colon, thyroid pancreas, gliomas, etc., highlighting its importance as a powerful molecular biomarker [19,20,21]. Furthermore, miR-21 enhances epithelial-mesenchymal transition and promotes early disease [22]. Its high expression levels induce therapeutic resistance in HER2+ (positive human epidermal growth factor receptor 2) breast cancer, which has been associated with a poor prognosis in patients with breast cancer [16]. Moreover, the overexpression of miR-21 is directly related to increased vascular endothelial growth factor (VEGF) expression levels and the formation of new blood vessels [23,24]. On the participation of miR-21 in apoptotic processes, high levels of expression of miR-21 have been inversely related to the expression of poly [ADP-ribose] polymerase 1 (PARP-1), an enzyme involved in the repair of damaged DNA. Therefore, in cancers in which miR-21 is overexpressed, the apoptosis process would be blocked by the inhibition of PARP-1 by miR-21 [25,26]. Hence, the downregulation of miR-21 could inhibit carcinogenic processes and improve the chemotherapeutic effect in breast cancer cells [21,27].

An miRNA sponge is a single-stranded mRNA molecule consisting of multiple tandem binding sites (MBS) that allow the capture of several miRNAs from the same family [28]. For this, the MBS contain a sequence called “seed”, which determines the specificity of miRNA binding to the sponge. This sequence is the same as the sequence that determines the specificity of miRNA binding to the 3’UTR end of its mRNA target [29,30,31]. Therefore, the seed sequence allows miRNA to bind specifically to the sponge.

One of the drawbacks of linear sponges is that they are susceptible to degradation by exonucleases. However, circular RNAs (CircRNAs), which lack 5′ ends and poly-A3′ structures, form continuous covalently closed RNA loops, making them more resistant than linear sponges to exonucleolytic RNA degradation [32]. The location of the Circ-RNAs is mainly cytoplasmic, although during the mitotic division they can be located in the nucleus as they are able to cross the pores formed by the rupture of the nuclear envelope in that process [31,32]. Regarding its synthesis, the exons that form the circ-NAs are usually surrounded by long introns enriched in ALU repetitions and these intronic elements are responsible for promoting the circulation by backsplicing [32,33,34,35]. 

The aim of this work was to demonstrate the use of a Circular sponge as a tool to capture miR-21, thus reducing its expression levels and, therefore, improve the chemotherapeutic effect on breast cancer cells, in addition to inhibiting carcinogenic processes and increasing apoptosis in breast cancer cells.

## 2. Results

### 2.1. MiR-21 Differential Expression in Breast Cancer Cells

**Figure 1:**
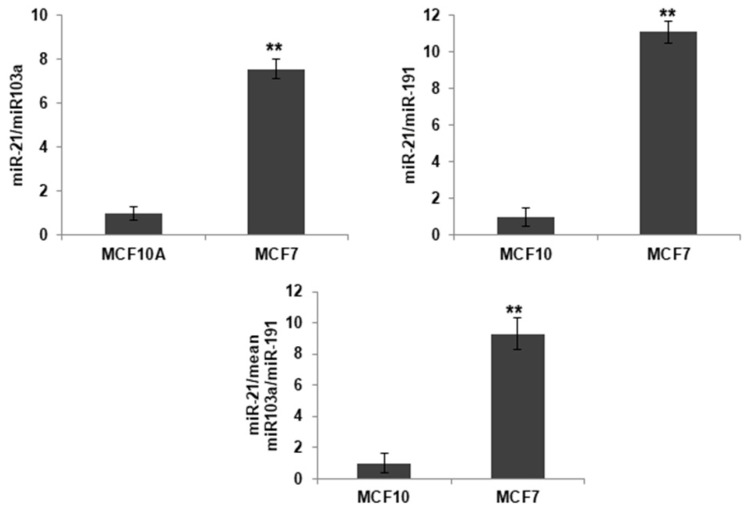
Basal expression of miR-21 in the MCF7 and the MCF10A cell lines. Relative expression levels of miR-21 were calculated upon miR-191 and miR-103 (house keeping genes) normalization, respectively, and mean expression value normalization of both miRNAs. Values represent means ± SD (*n* = 3). ** *p* < 0.01.

Mir-21 expression levels were determined in the MCF7 cells and the MCF10A cells using miR-103a and miR-191 like housekeeping. We used two housekeeping to increase the confidence of the results. Subsequently, we performed the normalization of the mean value of expression of both miRNAs. As expected, higher expression levels of miR-21 were detected in the MCF7 (breast cancer cell line) than in MCF10A (breast cell line). Based on this difference in the expression of mir-21, both lines were chosen to carry out the following assays and compare the results obtained in them. Statistical analysis was performed using a two-tailed *t*-test, comparing against the non-tumor line (MCF10A) (Figure 1).

### 2.2. Detection of Correct Expression of the Circular Sponge

**Figure 2:**
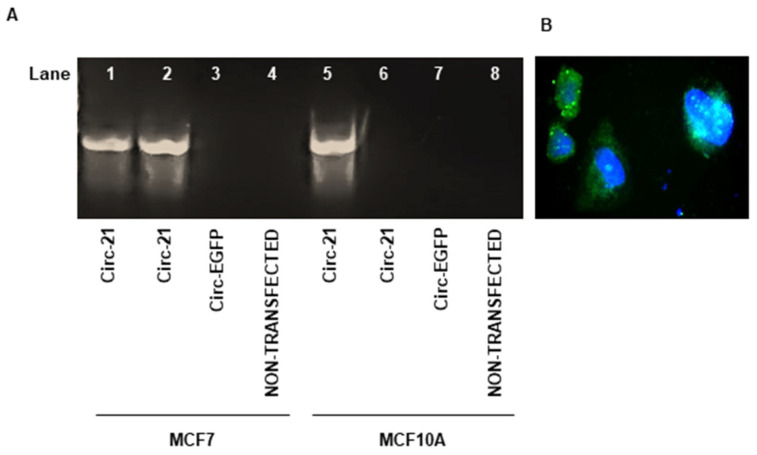
Expression of the Circ-21 by PCR (32 cycles). (**A**) Representative image of the PCR products. Lane 1, Circ-21 vector (positive control PCR); Lane 2 and 5, cells transfected with the Circ-21; Lane 3 and 7, cells transfected with the Circ-EGFP; Lane 4 and 8, non-transfected cells. The correct expression of miR-21 was confirmed in cells trasfected with the Circ-21. (**B**) Subcellular localization of the Circ-21. Representative microscopic image showing the detection of EGFP fluorescence (green) 72 h after transfection with the Circ-21 in MCF7. Cell nucleus were stained by DAPI (blue) (20×).

After transfecting both cell lines, the correct expression of the miR-21 sponge was determined by RT-PCR. cDNA was generated from 1 µg of RNA and the Circ-21 vector was used as a positive control for the PCR. As shown in Figure 2A, the Circ-21 transfected lines exhibited a band of the same size as that of the positive control, which was not observed in the Circ-EGFP transfected or in non-transfected cells. Furthermore, the Circ-21 expression was comparable in the MCF-7 cells (1.4-fold) and the MCF10A cells (1.5-fold), which allowed us to perform the following assays comparing both lines.

As described above in the methodology, the *EGFP* gene was used as a gene reporter. The detection of the expression of its fluorescent protein confirmed the correct expression of Circ-21 and helped to locate the Circ-EGFP expression in the cytoplasm by fluorescence microscopy (Figure 2B).

### 2.3. The Circ-21 Inhibits Breast Cancer Cell Growth 

**Figure 3:**
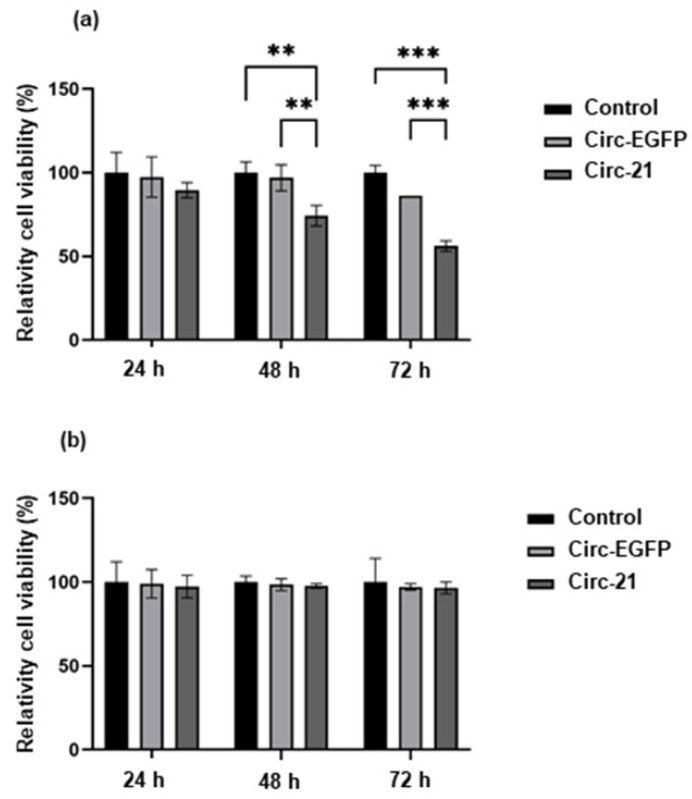
Effect of the Circ-21 on cell growth. MCF7 (**a**) and MCF10A (**b**) were transfected with the Circ-21 and the Circ-EGFP to determine proliferation rate modulation after 24, 48 and 72 h after transfection. The Circ-21 induced a time-dependent decrease in the MCF7 cell growth that was not observed when they were transfected with the Circ-EGFP or in the control cells Values represent means ± SD. (*n* = 3); ** *p* < 0.01 and *** *p* < 0.001.

As shown in Figure 3, the MCF7 cells transfected with the Circ-21 (Circ-21 MCF7) showed a significant and time-dependent decrease on cell growth, reaching a maximum at 72 h (43.84%; *p* < 0.001). These results were not observed in cells transfected with the Circ-EGFP (Circ-EGFP MCF7) or in non-transfected cells, whose cell growth values were similar. In addition, the MCF10A line (with low levels of miR-21) did not show decreased cell growth, whose cell growth values also were similar among the three groups. Statistical analysis was performed using a two-tailed *t*-test comparing the different samples against the control.

### 2.4. The Cir-21 Decreases Breast Cancer Cell Migration

**Figure 4:**
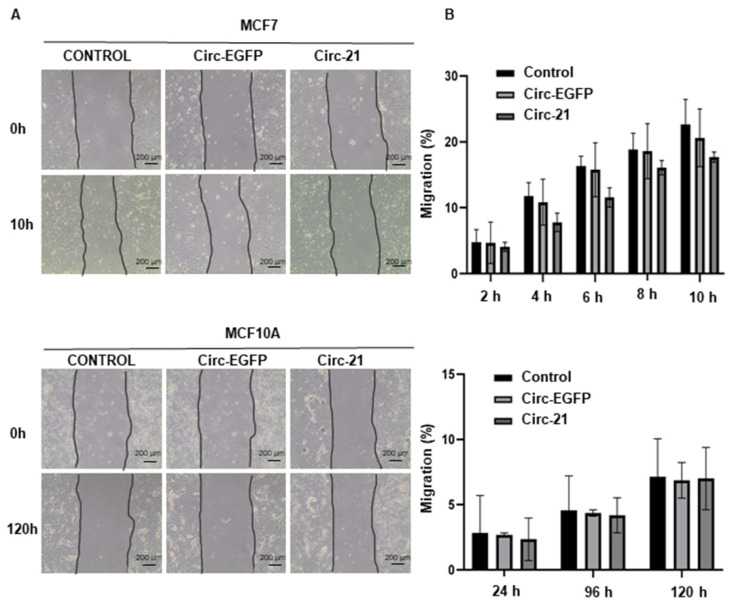
The Circ-21 inhibited breast cancer cell migration. (**A**) Representative microscopy images showing the wound-healing of the MCF7 and the MCF10A cells transfected with Circ-21, Circ-EGFP or no transfected; (**B**) Graphic representation of the percentage of the MCF7 and the MCF10A cells migration (area of the scratch) for the different conditions at different times. The least migration was observed in the Circ-21 MCF7 cells. Values represent means ± SD (*n* = 3).

Since miR-21 overexpression is related to tumor migration, we carried out the wound healing assay. By comparing the areas of the scratch, we were able to verify that the Circ-21 MCF7 cells showed a significant decrease in tumor cell migration vs. the Circ-EGFP MCF7 cells and non-transfected cells. By contrast, this reduction was not observed in the MCF10A cells (Figure 4). Wound healing assay times were different from MCF7 to MCF10A due to the cell growth characteristics. In both cases, the study was carried out until wound closure. Statistical analysis was performed using a two-tailed *t*-test comparing the different samples against the control.

### 2.5. Effect of the Circ-21 in Cell Colony Formation

**Figure 5:**
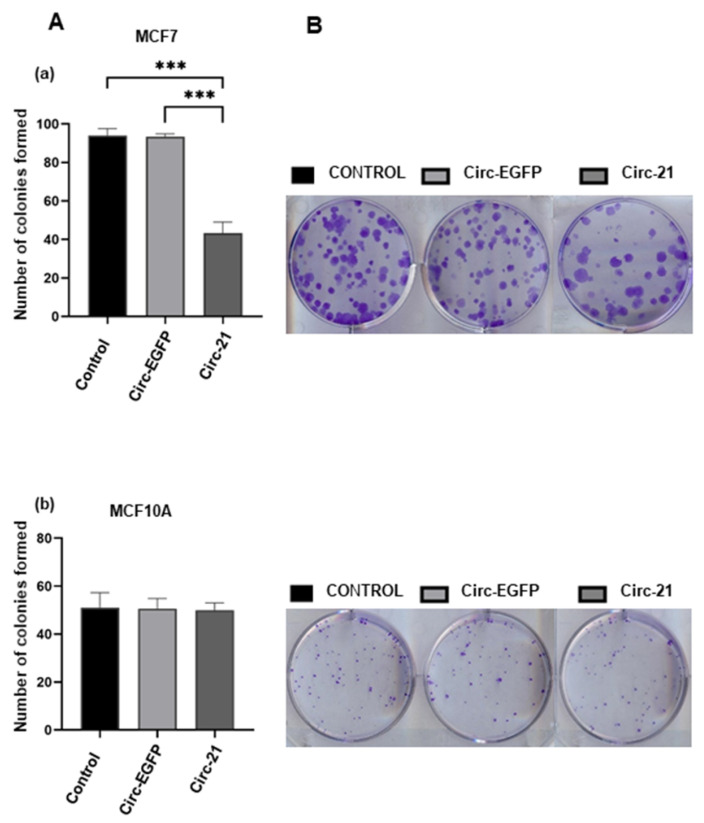
Effects of the Circ-21 on colony formation. (**A**) Circ-21, Circ-EGFP and control group of the MCF7 (**a**) and the MCF10A (**b**) cell lines were analyzed for 10 days by colony formation assay. The Circ-21 led to a decrease colony numbers in the MCF7 cells, but not in the MCF10A cells. Values represent means ± SD (*n* = 3). *** *p* < 0.001. (**B**) Representative image of colony formation.

The miR-21 overexpression leads to increased colony formation, so we studied how the Circ-21 could decrease this effect. The colonies formed were counted 10 days after the transfection, verifying that the Circ-21 MCF7 cells exhibited a lower number of colonies (43.3; *p* < 0.001) compared with the Circ-EGFP MCF7 cells (93.3) or control the MCF7 (94) (Figure 5). In contrast, no differences in the number of colonies were detected in the MCF10A cells for any of the three groups. Statistical analysis was performed using a two-tailed *t*-test, comparing the different samples against the control.

### 2.6. The Circ-21 Leads to Cell Cycle Arrest

**Figure 6:**
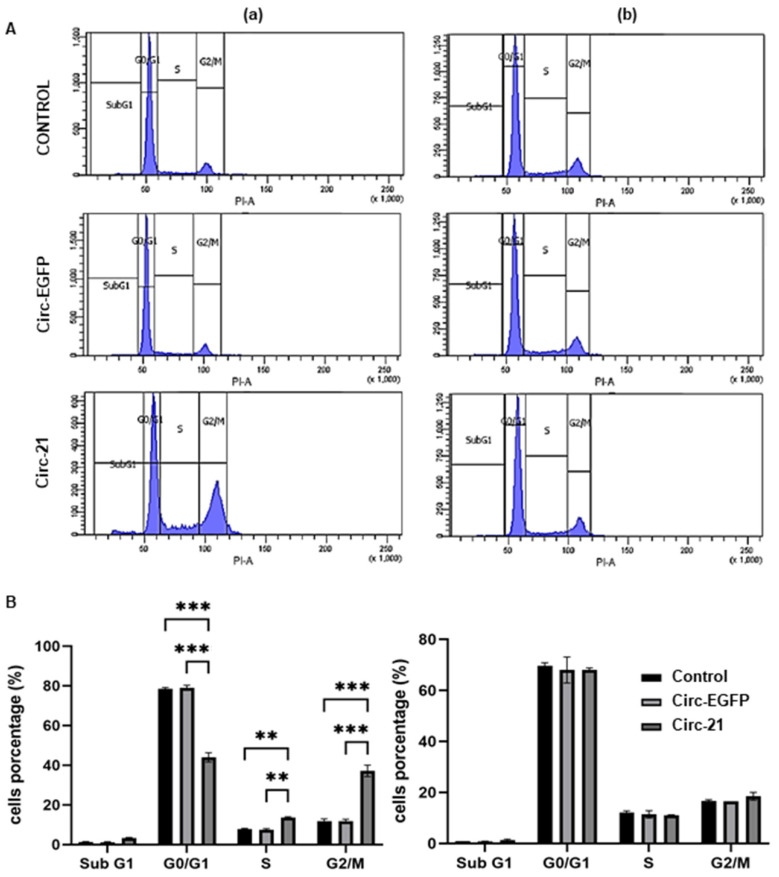
The cell cycle analysis of the MCF7 (**a**) and MCF10A (**b**). (**A**) Images of the FACScan flow cytometry results from cells exposed to PI/RNAse. (**B**) A graphic representation of percentage of labeled cells in each cell cycle phase. Cell cycle modification was only observed in the MCF7 cells, where the Circ-21 caused detection in G2/M. Values represent means ± SD (*n* = 2). ** *p* < 0.01 and *** *p* < 0.001.

We also studied how miR-21 sponging by the Circ-21 affects the cell cycle. As shown in Figure 6, the Circ-21 leads to cell cycle arrest in G2/M in the MCF7 cells. However, this was not observed in the Circ-EGFP MCF7 cells or in the non-transfected cells. Similar to the results described above, it was also not observed in the MCF10A cells for any of the three cases. Statistical analysis was performed using a two-tailed *t*-test, comparing the different samples against the control.

### 2.7. Western Blot

**Figure 7:**
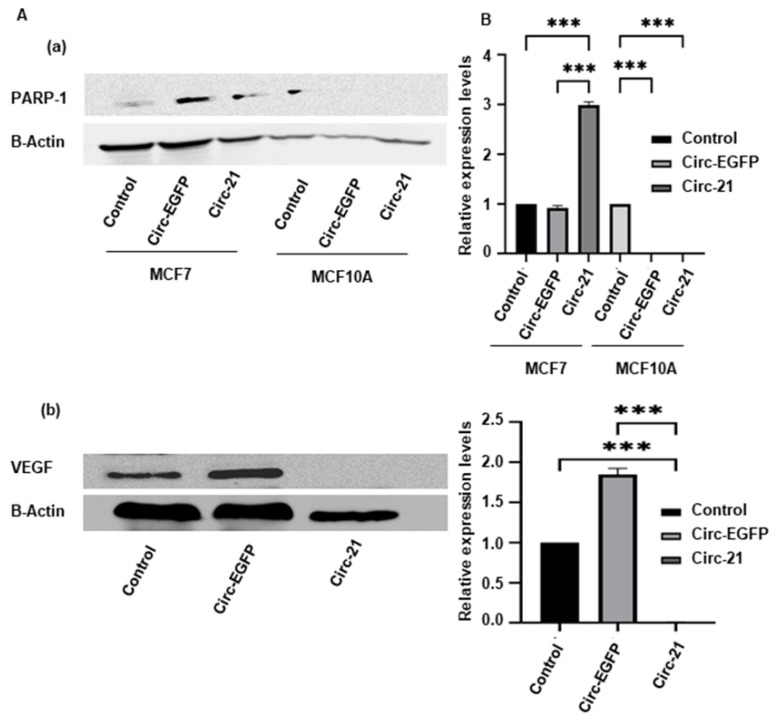
The Circ-21 actives PARP-1 (**a**) and decreases VEGF (**b**) expression in breast cancer cells. (**A**) A representative image of western blot analysis of PARP1 and VEGF. (**B**) A graphic representation of the densitometric analysis of PARP1 and VEGF. Relative expression levels represent PARP-1/beta-actin or VEGF/beta-actin ratios. Higher expression levels of PARP-1 in the Circ-21 MCF7 cells Circ-EGFP MCF7 cells and the control cells were detected. In contrast, no expression of VEGF was detected in the Circ-21 MCF7 cells. Values represent means ± SD (*n* = 3). *** *p* < 0.001.

Mir-21 has been inversely related to apoptotic processes, so we studied the expression of the PARP-1 protein, and this was directly related to tumor vascular increase, so we also studied the VEGF protein. Immunohistochemical analysis showed an increase in the expression levels of PARP-1 in the Circ-21 MCF7 cells vs the Circ-EGFP MCF7 cells and the control cells (*p* < 0.001). As expected, no expression levels were observed in the MCF10A (Figure 7A). In contrast, similar expression levels of VEGF were detected in the Circ-EGFP MCF7 cells and the control cells, but there was no expression in the Circ-21 MCF7 cells (*p* < 0.001) (Figure 7B). Statistical analysis was performed using a two-tailed *t*-test, comparing the different samples against the control.

### 2.8. Combined the Circ-21-Doxorubicin Therapy Induced Growth Disturbance on Breast Cancer Cells

**Figure 8:**
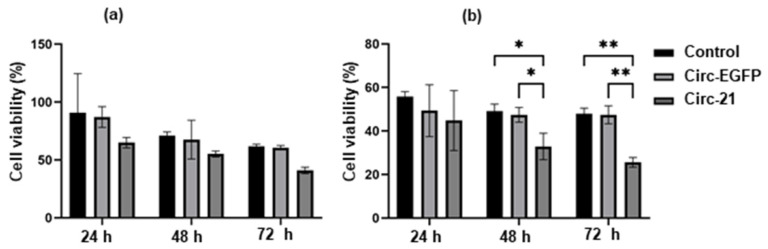
Effect of the combined therapy (DOXO + Circ-21) on MCF7 cell proliferation. DOXO concentrations of 0.02 µM (**a**) and 0.05 µM (**b**). The Circ-21 MCF7 cells treated with DOXO (Circ-21 + DOXO) showed a significant reduction in cell viability greater than with the separate treatments (only DOXO or only Circ-21). Values represent means ± SD (*n* = 3). * *p* < 0.1 and ** *p* < 0.01.

Once we established the effect of the Circ-21 on the breast cancer cell growth, we investigated its use in a combined therapy with (DOXO). After performing the IC50 (0.076 μM), we chose two concentrations close to and below the IC50 to perform the resistance test, which were 0.05 μM and 0.02 μM. The Circ-21 MCF7 cells treated with DOXO (Circ-21 + DOXO) showed a significant reduction in cell viability at 0.05 μM. This proliferation inhibition was time- and concentration-dependent, finding the greatest inhibition at 72 h and at the highest concentration (0.05 µM) (74.4%). This inhibition was greater than with the separate treatments, being 52.05% for the MCF7 treated with DOXO 0.05 μM and 43.84% for the Circ-21 MCF7 cells. This effect was not observed in the cells treated with DOXO + the Circ-EGFP or in non-transfected cells, where the effect observed was similar to that of DOXO treatment alone (Figure 8). Statistical analysis was performed using a two-tailed *t*-test comparing the different samples against the control.

### 2.9. Resitance Gene

**Figure 9:**
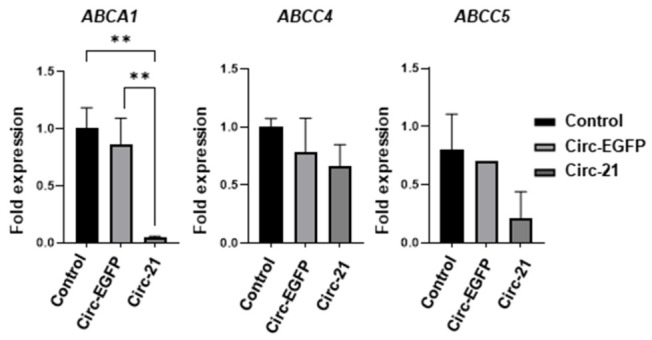
qRT-PCR analysis of drug resistance genes. The Circ-21 decreased the expression of *ABCA1*, *ABCC4* and *ABCC5* resistance gene. Values represent means ± SD (*n* = 3). ** *p* < 0.01.

Based on previous results in which we have demonstrated that sponge treatment increases the effects of DOXO, we studied the *ABCA1, ABCC4* and *ABCC5* resistance genes to look for a relationship between them and miR-21. As shown in Figure 9, all resistance genes exhibited a decrease in their expression levels in the Cic-21 MCF7 vs. non-transfected cells, with the *ABCA1* gene showing the greatest decrease. However, these differences were low and non-significant when the levels of the non-transfected cells vs. the Circ-EGFP cells were compared. Statistical analysis was performed using a two-tailed *t*-test, comparing the different samples against the control.

## 3. Discussion

Breast cancer is the most common cancer type in females and one of the leading causes of premature mortality in females worldwide [1]. Chemotherapy continues to be the main strategy for its treatment; however, multidrug resistance limits its effectiveness [2,4]. miRNA-21 (miR-21) is a microRNA that is upregulated in several types of cancer biomarkers [19,20,21], which has been related to drug resistance and carcinogenic processes [16]. Their high expression levels have been associated with a poor prognosis in patients with breast cancer [16]. We studied the expression level of miR-21 in the MCF7, breast carcinoma cells line, against the MCF10A, human normal breast cell line by qRT-PCR analyses, revealing that the expression levels of miR-21 were higher in the tumoral cell line. High levels of expression of mir-21 have also been detected in other lines of breast cancer [36]. Yan et al. [37], as well as ourselves, demonstrated the overexpression of miR-21 in the MCF7 line, the breast carcinoma cells line, against the MCF10A, a human normal breast cell line. Also, elevated levels of mir-21 have been detected in the plasma of breast cancer patients compared with healthy controls and benign breast tumor patients [38]. 

We have used a circular sponge targeted miR-21 (Circ-21) whose efficacy has previously been shown in lung cancer [39]. This sponge was designed containing seven tandem multiple linkers (MBS), based on hsa-miRNA-21-5P sequences (miRBase database), with a central bulge with four nucleotide mismatches at positions 10 to 13 of the MBS and with four nucleotide spacers between each MBS [30] which was inserted between the two flanking ALU sequences of pcDNA3.1(+) CircRNA Mini Vector. Circ-21 inhibited cell growth, decreasing migration, colony formation, and decreasing the volume of multicellular tumor spheroids in lung cancer cells. In contrast, normal lung cells were not altered. CircRNAs form continuous covalently closed RNA loops, making them more resistant than linear sponges to degradation by exonucleases [32]. For this reason, the latest studies are being carried out with circular sponges instead of linear ones. Karedath et al. [40] compared the ability of the pcDNA3.1(+) CircRNA mini vector to circularize DNA sequences introduced between its two ALU sequences against the pcDNA3.1 vector without Alu repeats, verifying that the former was the only one that could potentially circularize them. Xu et al. [41] also used pcDNA3.1(+) CircRNA mini vector to clone the complete circ_0005230 sequence to increase its expression in breast cancer cells, whose overexpression was related to adverse phenotypes in patients with this type of cancer. The pcDNA3.1(+) CircRNA mini vector has also been used to circularize hsa_circ_0001445 and hsa_circ_0001649, both downregulated in hepatocellular carcinoma. the overexpression of both circRNAs led to regulating the processes of proliferation, migration and invasion [42,43].

Overexpression of miR-21 is related to carcinogenic processes such as proliferation [44], migration [37,45], and colony formation [46]. Our results revealed that the Circ-21 induced a time-dependent decrease in the MCF7 cell growth that was not observed when they were transfected with the Circ-EGFP or in the control cells. No decrease in cell growth was observed in the MCF10A cells for any of the three cases. These results demonstrate the selective target of the Circ-21 to breast cancer cells, with high levels of miR-21 expression, thereby causing inhibition of their cell growth. Similar results have been reported after transfecting the MCF7 cells with an exogenous miR-21, showing increased proliferation after 24 h [47]. Huang et al. [44] observed a significant decrease in cell proliferation when transfecting the MCF7 cells with a chemically modified oligonucleotide specific to inhibit miR-21. These data corroborate that miR-21 is significantly related to proliferation in cancer cells. 

Similarly, less migration and colony formation was observed in the Circ-21 MCF7 cells compared with the Circ-EGFP MCF7 cells and the control cells. These results agree with Yang et al. [37], who observed a reduction in the migration of the MCF7 cells transfected with an anti-miR-21, but no variation was appreciated in the MCF10A cells. Similarly, the use of other inhibition of miR-21 suppressed cell proliferation, colony formation and migration in breast cancer cells, as well as proliferation and metastasis in vivo [38]. RNA nanoparticles loaded with anti-miR-21 suppressed triple-negative breast cancer cell invasion, migration, and colony formation [48]. 

miR-21 is also related to the cell cycle, which we have shown by observing a G2/M phase arrest only in the Circ-21 MCF7 cells. Zhong et al. [49] displayed cell cycle detection in G2/M phase in human lung cancer cells transfected with an anti-miR-21 inhibitor. However, the miR-21 overexpression decreased the percentage of cells in that phase and induced cycle detection in S phase. The use of hesperidin and luteolin downregulated the expression of miR-21 in the MCF7 and induced apoptosis, causing a significant accumulation of apoptotic cells into the G0/G1 and sub-G1 cell cycle phases [50]. Gao et al. [51] used a poly(L-lysine)-modified polyethyleneimine copolymer (PEI-PLL) to transport miR-21 sponge plasmid DNA (Sponge) or anti-miR-21 oligonucleotide (AMO) into the MCF7 cells. Both PEI-PLL groups exhibited decreased cell viability and cell cycle arrest in G1 phase.

Our results relate this apoptosis with the activation of PARP-1. We detected an overexpression of PARP-1 in the Circ-21 MCF7 cells that was not observed in the Circ-EGFP MCF7 cells or in the control cells. Zhang et al. [25] conclude that by treating human bronchial epithelial cells with miR-21 mimics, PARP-1 expression was significantly inhibited; however, when cells were treated with a miR-21 inhibitor, PARP-1 expression levels were markedly elevated. Therefore, we can affirm that miR-21 negatively regulates PARP-1 [26], thus inhibiting apoptosis, and that our sponge is capable of inhibiting the expression of mir-21, thus inducing apoptosis by activating PARP-1. 

To determine the relationship between miR-21 and the formation of new blood vessels, we studied the angiogenic growth factor VEGF. While the Circ-EGFP MCF7 cells and control cells showed expression of VEFG, the Circ-21 MCF7 cells did not show expression of this factor. On the other hand, VEGF was not studied in the MCF10A line because it is a non-tumor line and does not present angiogenic growth. These data demonstrate that our Circ-21 reduces the malignancy of the cells by not forming new blood vessels to nourish the tumor. Similar results have been described in gastric cancer cell lines, where miR-21 upregulation has been associated with increased VEGF expression levels [23]. Sun et al. [24] demonstrated in the PANC-1 pancreatic cancer line that silencing miR-21 inhibited VEGF expression. 

Finally, we studied how the Circ-21 affected the resistance of the MCF7 tumor cell line to DOXO treatment. Our results determine that the combination of the Circ-21 with DOXO (Circ-21+DOXO) enhances the inhibition of tumor cell proliferation in comparison to single treatments, suggesting that such a combined therapy could reduce the effective drug dose in patients, and therefore the resulting side effects. Wang et al. [27] reported similar results. They studied the breast cancer cell lines MCF7 and MCF7/ADR (resistant to DOXO), showing that the IC50 of the ADR cells transfected with the inhibitor miR-21 was lower than the IC50 of the cells of control. Zhang et al. [52] developed three self-assembling DNA nanosponges (DNS). One contained miR-21 tandem binding sites for removal (DNS/miR-21). Another contained double chain DOXO binding sites (DNS/DOXO). A third contains both assemblies (DNS/DOXO/miR-21). All of them also contained the complementary sequence to the MUC1 aptamer for binding to the surface of tumor cells. The results determined that DNS had a higher affinity for tumor cells (MCF7) than for normal cells (Hs578 Bst). Furthermore, DNS/miR-21/DOXO caused >80% cell death, compared to 50% with DNS/DOX in the MCF7 cells.

Jung et al. [53] used a multipotent miRNA sponge capable of inhibiting several miRNAs simultaneously, including miR-21, in combination with DOXO in breast and pancreatic cancer cells. The results agree with ours, revealing that the miRNA sponge sensitizes cells to cancer DOXO, since the combined treatment decreased cell proliferation more than when cells were treated with DOXO alone. In addition, cell migratory activity was attenuated.

Likewise, the decrease in the expression of miR-21 could improve therapeutic efficacy, increasing the effect of other chemotherapeutic agents such as taxol [21].

After demonstrating the increase in sensitization to DOXO of the MCF7 cells due to treatment with our Circ-21, we decided to study the drug resistance genes *ABCA1*, *ABCC4* and *ABCC5*, verifying that in the Circ-21 MCF7 cells the expression of these genes decreased compared to the Circ-EGFP MCF7 cells or the control cells. These data would explain the enhanced effect of DOXO observed in the Circ-21 MCF7 cells. Corroborating our data, previous studies have shown that the MCF7 cell line has a significant expression of genes of the ABC family such as *ABCC1* and *ABCC4*. In addition, higher levels of expression of the *ABCC4*, *ABCC5* and *ABCB1* genes have been detected in the MCF7/ADR cells than in the MCF7 cells [54,55]. The expression of *ABCB1* has been related to the overexpression of miR-21 in the MCF7 cells, achieving that, after treating the cells with anti-miR-21, its expression is reduced [56]. The expression of *ABCC1*, *ABCC2*, *ABCC4* and *ABCC5* were measured by qRT-PCR in the resistant MCF7/ADR and MCF7. All of them were upregulated in the MCF7/ADR cells compared to the MCF7, with ABCC5 (5.21-fold) showing the greatest increase [55]. All these results confirm the important role of miR-21 in resistance to chemotherapy.

## 4. Materials and Methods

### 4.1. Cell Culture

Breast carcinoma cells (MCF7) and human normal breast cells (MCF10A) were obtained from the Instrumentation Service Center (CIC, University of Granada, Granada, Spain). The tumoral cell line was grown in Dulbecco’s Modified Eagle’s Medium (DMEM) (Sigma-Aldrich, Madrid, Spain), supplemented with 10% fetal bovine serum (FBS), while the non tumoral cell line was grown in DMEM/F12 medium supplemented with 10% horse serum, 20 ng/mL EGF, 10 μg/mL insulin, and 50 μg/mL hydrocortisone. Both were also added to 1% streptomycin-penicillin (Sigma-Aldrich) and cultured under air containing 5% CO_2_ and in an incubator at 37 °C.

### 4.2. miR-21 Expression Levels

To determine expression, RNA was extracted from both cell lines with the miRCURY LNA miRNA PCR Starter Kit (Qiagen). To carry out the quantitative real-time polymerase chain reaction (qRT-PCR), we followed the protocol described by Rama et al. [31] using miR-103a and miR-191 using miR-103a and miR-191 as housekeeping. 

### 4.3. Sponge Expression Vector 

We used the pcDNA3.1(+) CircRNA mini vector (Addgne), in which a sponge against miR-21 (Circ-21) had been subcloned, and on the other hand, the *enhanced green fluorescent protein* (*EGFP*) gen (Circ-EGFP), which was used as the reporter. Both of them were designed and constructed by Rama et al. [31]

### 4.4. Transfection of Cells

Before carrying out the transfection process, the MCF7 and MCF10A cells were seeded in their respective culture medium and incubated overnight. Lipofectamine 2000 (Invitrogen) was used to develop the transfection of the Circ-21 and the Circ-EGFP following the instructions of the manufacturer, obtaining three experimental groups: cells transfected with the Circ-21, cells transfected with the Circ-EGFP and control cells (cells not transfected).

### 4.5. Detection of the Circ-21 Expression

RT-PCR was used to determine the sponge expression. 24 h after transfection, RNeasy Mini kit (Qiagen) was used for the extraction of RNA from different group of transfections (Cir-21, Circ- EGFP and non-transfected). From 1 µg of RNA, cDNA was generated following the protocol of Promega Reverse Transcription System (Promega, Madrid, Spain) using total cellular RNA (1 µg). After that, Polymerase chain reaction (PCR) amplification of the *EGFP-miR-21* sponge was carried out for 32 cycles to determine correct sponge expression. Briefly, the Circ-21 vector (miR-21 subcloned into pcDNA3.1(+) CircRNA) was used as a positive control. The PCR products were separated by agarose gel electrophoresis (1.5%) and visualized with RedSafe Nucleic Acid Stain Solution (iNtRON Biotechnology). The Bio-Rad documentation system (Quantity One Analysis Software 4.6.6) was used to obtain and quantify the images.

Also, microscopic analysis was used to corroborate EGFP expression, which was used as the reporter above. To stain cell nuclei, 100 nM DAPI (Invitrogen) was used. EGFP was excited at 488 nm and DAPI nuclear stain at 364 nm. Fluorescence microscopy analysis was carried out with a Leica DMI6000 microscope (Heidelberg, Germany).

### 4.6. Cell Proliferation Assay

To perform the cell proliferation assay, 3 × 10^3^ the MCF7 cells/well and 6 × 10^3^ MCF10A cells/well were seeded in 48-well plates and transfected as described above. MTT (3-(4,5 dimethylthiazol-2-yl)-2,5-diphenyltetrazolium bromide) solution (5 mg/mL) was added to each well (10 μL) and incubated for 4 h at 37 °C. After incubation time, medium was removed and 100 µL of dimethylsulfoxide (DMSO) were added to each well. Optical density was determined using a Titertek multiscan colorimeter (Flow Laboratories, Oldham, UK) at 570 and 690 nm. The proliferation effect of miR-21 was determined at 24, 48 and 72 h after transfection. All experiments were performed in triplicate.

### 4.7. Wound Healing Assay

In vitro migration assay was performed to determine the tumor cell migration capacity of cells. For that, MCF7 (2 × 10^5^ cells/well) and MCF10A (3 × 10^5^ cells/well) were seeded in 12-well plates and transfected as described above. When confluence was 90%, a “wound” was carried out using a sterile 100 pipette tip following Grada et al. [57] After that, medium without FBS replaced the previous medium. The monitoring of cell migration was performed by taking images at different times depending on the migration speed of each cell line using a Leica microscope (Wetzlar, Germany). The results were analyzed using MRI Wound Healing Tool of ImageJ software (version 1.52s) (National Institutes of Health, Bethesda, MD, USA, https://imagej.nih.gov/ij/ (accessed on 5 April 2022)). All experiments were performed in triplicate. Migration was calculated using the following formula:

Migration (%) = 100 − ((area of the wound time X/area of the wound time 0) × 100)

### 4.8. Colony Formation

The MCF7 and MCF10A cells were seeded in 12-well plates (400 cells/well) and transfected as described above. One week after seeding, medium was removed, after fixing the cells, the colonies were stained with 1 mL/well of 0.5% crystal violet in 70% methanol during 15′. Finally, after being dried overnight, the number of colonies was determined. All experiments were performed in triplicate.

### 4.9. Cell Cycle Analysis

To perform the cell cycle analysis, 35 × 10^3^ the MCF7 cells/well and 5 × 10^4^ MCF10A cells/well were seeded in 12-well plates and transfected as described above. 72 h after transfection, medium was collected and adherent cells were harvested by trypsin-EDTA solution to produce a single cell suspension. Then, cells were pelleted by centrifugation, fixed in 70% ice cold ethanol and stored at −20 °C. When ethanol was removed, cells were processed using the PI/RNAse Solution Kit (Immunostep, Salamanca, Spain) for 20 min. The stained cells were analyzed by FACScan flow cytometer (Becton Dickinson, San Jose, CA, USA) using FlowJo software v10.2 (Treestar, Ashland, OR, USA), determining the phase of the predominant cell cycle. The experiments were done in duplicate.

### 4.10. Western Blot Analysis

Western blot analysis was carried out by proteins obtained from transfected MCF7 and MCF10A cells during 72 h. After extracting the proteins from the cells with RIPA (Ra-dio-Immunoprecipitation Assay) lysis buffer (Thermo Fisher Scientific, Waltham, MA, USA), they were quantified using Bradford. To perform the western blot, 40 μg of protein from each sample was loaded on a 10% SDS-PAGE gel in a Mini Protean II cell (Bio-Rad, Hercules, CA, USA). After the transfer process, the membranes were incubated for 1 h with blocking solution (phosphate-buffered saline (PBS)-Tween-20 at 0.1% + milk powder at 5% (*w*/*v*)). They were then incubated with the primary antibody overnight at 4 °C (anti-PARP-1 polyclonal rabbit immunoglobulin G (IgG) (sc-8007), 1:1000 dilution; anti-VEGF (sc-7269), 1:1000 dilution (Santa Cruz Biotechnology, Santa Cruz, CA, USA). After incubation with peroxidase-conjugated secondary antibody (1:5000 dilution) (Goat anti-mouse IgG-HRP, Santa Cruz Biotechnology) was revealed. Anti-β-actin IgG (A3854, Sigma-Aldrich) (dilution 1:10,000) was used as internal control. Finally, the bands obtained were analyzed using the analytical software Quantity One 4.6.6 (Bio-Rad).

### 4.11. Combined Treatment

Previously, the IC50 for DOXO (Sigma-Aldrich) was calculated. For this, 4 × 10^3^ cells/well of the MCF7 line were seeded in 48-well NUNC® plates and increased concentrations of DOXO were used, 0.01 μM, 0.02 μM, 0.05 μM, 0.1 μM, 0.2 μM, 0.5 μM, 1 μM, 3 μM. A control group, without drugs, was also included. The effect of the combined therapy of Circ-21 with DOXO (DOXO + Circ-21) was carried out under the same conditions using 0.05 μM and 0.02 μM DOXO. The cells treated with DOXO the Circ-EGFP and no treated cells (control) were also studied. The assay was performed in triplicate and at times of 24, 48 and 72 h.

### 4.12. RNA Extraction and Quantitative Real-Time PCR

Total RNA from the MCF7 cell line, transfected as described above, was extracted with Trizol Reagent (RNeasy Mini Kit, Qiagen, MD, USA) and cDNA was generated by means of the MMLV-RT (Promega, Madison, WI, USA) using a retro-transcriptase kit following the manufacturer’s instructions.

Real-time PCR was performed using SYBR Green Supermix (Taq Universal SYBR Green Supermix) (Bio-Rad). Quantitative RT-PCR primers and hybridization temperatures (Tm) specific for the genes tested (ABCA1, ABCC4, and ABCC5) are listed in Supplementary Materials Table S1. Glyceraldehyde-3-phosphate dehydrogenase (*GAPDH*) was used as endogenous, normalizing the gene expression data with it and calculating the relative expression levels applying the 2−∆∆Ct method. All quantitative RT-PCR assays were performed on an ABI 7900 system (ABI). The experiments were done in triplicate.

### 4.13. Statistical Analysis

The obtained results were expressed as the mean ± standard deviation (SD). To carry out the statistical analysis, the Statistical Package for the Social Sciences (SPSS) v. 15.0 was used with the Student’s *t*-test, comparing the treatments with the control with a significance level of 0.1 (*p* = 0.1).

## 5. Conclusions

We demonstrated that our Circ-21 is an excellent sponge capable of inhibiting cell growth, decreasing migration and colony formation in breast cancer cells. Besides, our Circ-21 caused cell cycle arrest in G2/M phase induced by PARP-1 activation, and in addition, VEGF decreased, which reduces the malignancy of the breast cancer cells by not forming new blood vessels to nourish the tumor. The combined use of our Circ-21 with DOXO potentiates the effect of this drug compared to individual treatments, suggesting that this combination therapy could reduce the effective dose of DOXO in patients and thus the resulting side effects. This increase in DOXO sensitization of breast cancer cells could be due to the decreased expression of genes related to drug resistance, caused by our Circ-21.

These results suggest that miRNA silencing by circular sponges may be considered as a powerful tool in the field of gene therapy and, in particular, that the sponge against miR-21 is an effective candidate for cancer treatment. 

## Data Availability

Not applicable.

## Supplementary Materials

The following supporting information can be downloaded at: https://www.mdpi.com/article/10.3390/ijms232314803/s1.
