# Supplementary material for: Circular Sponge against miR-21 Enhances the Antitumor Activity of Doxorubicin against Breast Cancer Cells
Source: Int J Mol Sci. 2022 Nov 26;23(23):14803. doi: 10.3390/ijms232314803 (PMC9736351; doi:10.3390/ijms232314803)
Supplement: Supplementary file 1 [file ijms-23-14803-s001.zip › ijms-1964758-supplementary.pdf]

Supplementary Materials Table S1

Table S1. Primers used to perform qPCR.

Gene	Sequence
GADPH-F	TGCACCACCAACTGCTTAGC
GADPH-R	GGCATGGACTGTGGTCATGAG
ABCA1-F	GCACTGAGGAAGATGCTGAAA
ABCA1-R	AGTTCCTGGAAGGTCTTGTTCAC
ABCC4-F	CCATTGAAGATCTTCCTGG
ABCC4-R	GGTGTTCAATCTGTGTGCA
ABCC5-F	GGGATAACTTCTCAGTGGGG
ABCC5-R	GGAATGGCAATGCTCTAAAG
